# Evaluation of Tensile Strength of Surgically Absorbable Suture Materials Used in Oral Surgery after Immersion in Different Beverages: An In Vitro Study

**DOI:** 10.3390/ma17143586

**Published:** 2024-07-20

**Authors:** Ahmet Aktı, Doğan Ilgaz Kaya

**Affiliations:** 1 Department of Oral and Maxillofacial Surgery, Dentistry Faculty, Selçuk University, Konya 42250, Turkey; 2 Department of Oral and Maxillofacial Surgery, Dentistry Faculty, Karamanoğlu Mehmetbey University, Karaman 70100, Turkey; dt.doganilgaz@gmail.com

**Keywords:** suture material, tensile strength, oral surgery, beverages, dentistry

## Abstract

Suture materials are natural or synthetic biomaterials used to close tissues together. After surgical procedures in the mouth, the surgical site and the sutures are physically affected by many different factors. This study was conducted to evaluate the effects of frequently consumed beverages on the tensile strength of monofilament PGLA (polyglycolide-co-l-lactide) and multi-filament PGCL (polyglycolide-co-caprolactone) absorbable sutures. In particular, PGLA and PGCL absorbable sutures, which are frequently used in oral surgery, were used to evaluate the change in the strength of suture materials. The suture materials were soaked in tea, coffee, and cola drinks five times a day for 5 min each and the rest of the time in artificial saliva. All suture materials were aged via thermal cycling. Tensile strengths were tested at 0, 3, 7, and 14 days. Mixed ANOVA (four replicates: within-group comparison and two factors: between-group comparison) was performed to evaluate the effects of groups and time on the tension levels of the Tekmon and Vicryl suture materials. Analysis of Variance was used for the within- and between-group comparisons, with the Bonferroni corrected *t*-test for multiple comparisons. For the PGCL suture material, there were significant decreases in tension levels in artificial saliva, tea, coffee, and cola at time T3 compared to T0, T1, and T2, and at T1 and T2 compared to T0. For the PGLA suture material, there were significant decreases in tension levels in artificial saliva, tea, coffee, and cola at time T3 compared to T0, T1, and T2. There was also a significant decrease in tension level at time T2 in cola compared to T0. The present study demonstrates that beverages significantly decrease the strength of suture materials for 14 days after surgery. In particular, cola decreased the resistance of the PGCL suture material more significantly in the first week when compared to other beverages.

## 1. Introduction

Suture materials are natural or synthetic biomaterials used to allow tissues to close. The goals of wound closure are to eliminate dead space, evenly distribute tension along deep suture lines, and maintain tensile strength across the wound until the tissue’s tensile strength is sufficient for permanent closure [1]. The use of suture materials for wound closure dates back to ancient Egyptian times, as evidenced by inscriptions dating to 3500 BC [1].

Wounds generally heal in two ways: primary or secondary. Primary healing involves the healing of the same tissues through regeneration surgery, while secondary healing refers to healing in which the injured tissues do not regenerate but are repaired or replaced. The most important factor in primary healing is suturing. In this approach, it is important that the suture remains intact until the end of the healing process, in order to bring the two wound edges together permanently. Different environments affect the strength of sutures. For example, when a suture is placed in the mouth, part of it is in the tissue and resists hydrolytic and proteolytic enzymes, while the other part resists bacteria in saliva and oral flora [1,2].

Suturing in dentistry differs from suturing in other parts of the human body due to the type of tissue involved, the constant presence of saliva, increased blood supply to the tissue, as well as functions such as speaking, chewing, and swallowing [3]. Suture materials are regularly exposed to mechanical forces in the mouth during eating, speaking, and making facial expressions, as well as different pH levels in the oral environment and proteolytic enzymes produced by salivary bacteria. The most important feature of an ideal suture material is its ability to close wounds with minimal stress or no strain, allowing for optimal healing [4,5].

In the oral environment, temperature and pH are constantly changing due to nutritional factors. After surgical procedures in the mouth, the surgical site and sutures are directly affected by these temperature and pH changes. In a study conducted by Hans et al., a statistically significant difference in mean salivary pH was observed prior to and after the consumption of cola, fruit drinks, coffee, and sweetened milk at varying time intervals [6]. Compared to solid foods, liquid beverages seem to have a shorter clearance time, but maintain a low pH level for a longer period of time. The suture material loses its tensile strength more quickly in the oral environment due to such variables, which may lead to less tissue adaptation and a higher susceptibility to secondary infections [7]. 

Suture materials can be divided into resorbable and non-resorbable sutures, according to their degradability. The primary advantage of resorbable sutures is the absence of patient discomfort due to suture removal and the absence of an additional visit to the clinic. Not removing the suture increases patient comfort during the healing process through eliminating the associated pain and anxiety. Patients can focus on their recovery without the added burden of waiting for suture removal appointments. These advantages are among the reasons why absorbable suture materials are preferred over non-resorbable suture materials. PGCL (polyglycolide-co-caprolactone) is an absorbable monofilament suture. It contains poly(glycolide-co-caprolactone) (PGCL), homo-polyglycolide (PGA), and polycaprolactone (PCL). Due to its synergistic properties such as excellent bioresorption and flexibility obtained from these ingredients, it is widely used as an absorbable suture in the context of oral surgery [8]. PGLA (polyglycolide-co-l-lactide) is an absorbable multi-filament suture, first marketed in 1974 as a copolymer of glycolic acid that contains 90:10 units of glycolic acid per lactic acid. PGLA sutures require multi-strand preparation, and have the characteristics of fast absorption and protection [9]. 

This study was conducted to evaluate the effect of frequently consumed beverages on the tensile resistance of monofilament PGLA and multi-filament PGCL absorbable sutures. At the same time, we attempted to create the most suitable environment for the oral environment through the use of a thermal aging process.

## 2. Materials and Methods

The in vitro study described in this paper was conducted in the Research Laboratory of the Faculty of Dentistry, Selçuk University, between February 2024 and April 2024. PGLA (polyglycolide-co-l-lactide) and PGCL (polyglycolide-co-caprolactone), which are absorbable sutures frequently used in oral surgery, were used to evaluate the changes in the strength of the suture materials. Four different solutions were used to evaluate the physical properties of the suture materials. Artificial saliva was used as a control and coffee (Nescafe Classic, Nestle, Vevey, Switzerland), tea (Çaykur Black Tea, Rize, Turkey), and cola (Coca Cola Company, Atlanta, GA, USA) were used to evaluate the tensile strength of the sutures in different beverages. The beverages used were planned similarly to the study of Abullais et al. [10].

### 2.1. Preparation of Solutions

The recipe described by Alsarhan et al. was used to prepare artificial saliva [11]. In brief, 100 mL each of 25 mM potassium phosphate (K_2_HPO_4_), 24 mM sodium phosphate (Na_2_HPO_4_), 1.570 mM potassium bicarbonate (KHCO_3_), 100 mM sodium chloride (NaCl), and 1.5 mM magnesium chloride (MgCl_2_) were mixed, following which 6 mL of 25 mM citric acid (C_6_H_8_O_7_) and 100 mL of 15 mM calcium chloride (CaCl_2_) were added. The solution and samples were kept in a 37 °C incubator prior to and during the experiment.

Tea and coffee were prepared via brewing using traditional methods. Their temperatures were allowed to drop below 40 °C before being used in the experiment. The cola was stored in previously unopened packaging and at room temperature.

3-0 PGLA (Pegelak, Doğsan, Trabzon, Turkey) and 3-0 PGCL (Tekmon, Doğsan, Trabzon, Turkey) were used as suture materials. Each suture material was tested on days 0, 3, 7, and 14. Test times were planned similar to the study by Abullais et al. [10].

**Figure 1:**
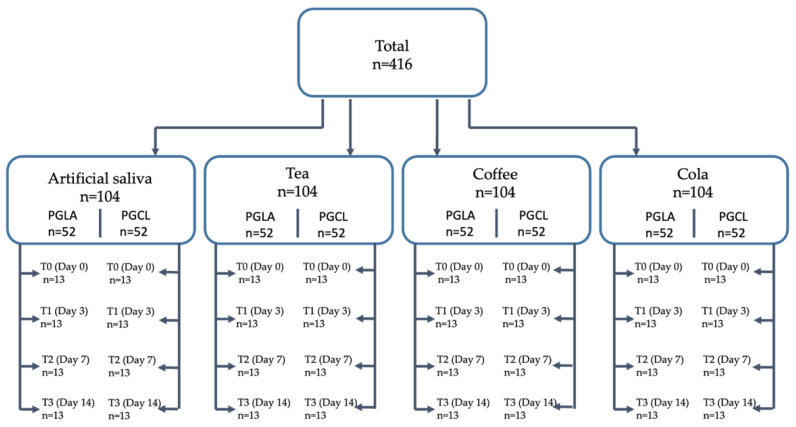
Flowchart of the study.

In total, 416 suture materials were included in the study in 8 different groups, with 52 specimens in each group (Figure 1). Simple sutures were applied, with 5 knots in each specimen. The dimensions of all knotted samples were the same length. The specimens in the beverage groups were exposed to the test solutions 5 times every day for 5 min, then washed with saline and placed back in artificial saliva, similarly to the studies of Abullais et al. [10]. All specimens were kept in artificial saliva until the final test.

### 2.2. Thermal Cycling Process

The thermal cycling temperature was set as 5–55°, based on the recommendations of the International Standards Organization (ISO) published in 2015 [12]. There is no consensus on the number of times that the temperature changes in the mouth environment on a daily basis. Many researchers have suggested that 10,000 thermal cycles correspond to 1 year of clinical function. This estimate is based on the hypothesis that such cycles may occur 20–50 times a day and is accepted by many authors [13,14]. Therefore, we decided to use 40 cycles per day. We set the saliva immersion time to 50 s, such that temperature changes could completely pass through the plastic tube and saliva could affect the suture materials. The aim was to physically age the samples in a thermal cycling device (Dental Teknik, Thermal Cycle, Istanbul, Turkey).

**Figure 2:**
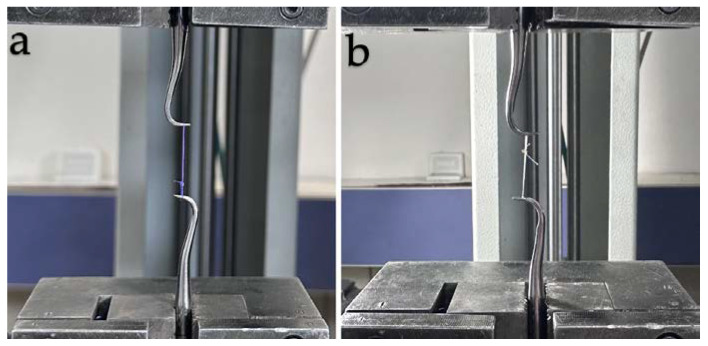
Images of the suture materials on the Instron pulling system. (**a**) PGLA, (**b**) PGCL.

The mechanical properties of the suture materials were tested using a Universal Testing Machine (Instron Testing System Model 5965, Norwood, MA, USA). The tensile strength of the suture specimens was evaluated at baseline (i.e., day 0) and at days 3, 7, and 14 after immersion in the test environment. Each specimen was attached to two metal hooks located on opposite arms of the machine (Figure 2). A pilot test was performed to demonstrate that this experimental setup did not lead to a failure of the hooks or knots. The samples were stretched with the help of a hook and pulled at a speed of 5 mm/min until they broke. The value at the moment of breakage was recorded as the maximum tensile strength in Newtons.

## 3. Statistical Analysis

In the power analysis of this study, it was determined that the study should be performed with at least 352 samples in total for Mixed ANOVA to be performed at a 5% significance level, 80% statistical power, and 0.25 (moderate) effect size. All statistical analyses were performed using the statistical software language R version 4.1.2 (The R Foundation for Statistical Computing, Vienna, Austria; https://www.r-project.org (accessed on 1 November 2021). Before the analyses, the normality of the data were checked with the help of the Shapiro–Wilk normality test and Q–Q graphs, and the assumption of sphericity was checked with the Mauchly test. The findings of the numerical variables in the study are presented as the mean ± standard deviation. Mixed ANOVA (4 replicates: within-group comparison and 2 factors: between-group comparison) was performed to evaluate the effects of groups and time on the tension levels of the Tekmon and Vicryl suture materials. An Analysis of Variance was used for within- and between-group comparisons, with the Bonferroni corrected *t*-test for multiple comparisons. The statistical significance level was determined as 0.05.

## 4. Results

The effect of measurement times on tension levels was statistically significant (F = 758.710, *p* < 0.001, =0.888). The interaction between measurement times and suture materials was statistically significant (F = 139.091, *p* < 0.001, =0.592). The interaction between measurement times and fluids was statistically significant (F = 4.978, *p* < 0.001, =0.135). The effect of the interaction between measurement times, suture materials, and fluids on tension levels was also statistically significant (F = 3.404, *p* < 0.001, =0.096).

The main effect of suture materials on tension levels was significant (F = 1212.529, *p* < 0.001, =0.927), as well as the main effect of fluids on tension levels (F = 24.741, *p* < 0.001, =0.436). On the other hand, the effect of the suture–fluid interaction on tension levels was not statistically significant (F = 2.017, *p* = 0.117, =0.059).

**Figure 3:**
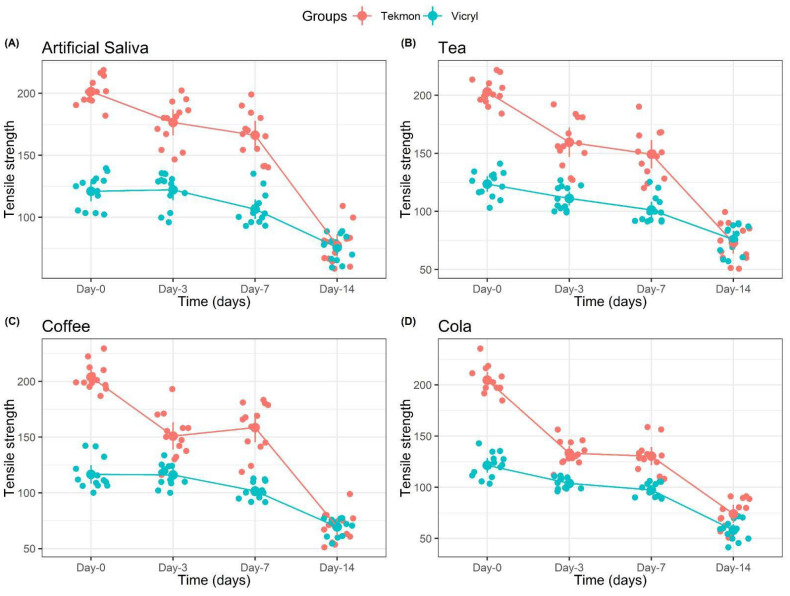
Comparison of tension levels between suture materials in (**A**) artificial saliva, (**B**) tea, (**C**) coffee, and (**D**) cola (Tekmon: PGCL, Vicryl: PGLA).

Comparisons of the tensile strength levels of PGCL and PGLA suture materials, according to the fluids at each measurement time, are given in Table 1. Graphical comparisons of the tension levels of PGCL and PGLA suture materials, according to the fluids at each measurement time, are shown in Figure 3.

For the PGCL suture material, there was a significant decrease in tension levels at times T1 and T3 in all solutions.

For the PGLA suture material, there was a significant decrease in tension levels at time T3 in all solutions. There was also a significant decrease in tension levels at time T2 in cola.

For the PGCL suture material, the tension levels at T1 and T2 were significantly lower in coffee and cola compared to artificial saliva. On the other hand, there was no difference between the tension levels in liquids at T0 and T3 (all *p* > 0.05).

For the PGLA suture material, there was no difference between the tension levels in the fluids at T0, T1, T2, and T3 (all *p* > 0.05).

At T0, T1, and T2, the tension levels of the PGLA suture materials measured in artificial saliva, tea, coffee, and cola were significantly lower than those for the PGCL suture materials.

At T3, the tension levels measured in artificial saliva, tea, coffee, and cola were similar in the PGCL and PGLA suture materials (all *p* > 0.05).

## 5. Discussion

Appropriate sutures require specific physical characteristics and properties, such as good tensile strength, dimensional stability, lack of memory, knot security, and sufficient flexibility to avoid damage to the oral mucosa [4]. Therefore, the selection of suture materials with high physical strength is important for proper wound healing.

In this study, we used PGLA, a resorbable multi-filament suture, and PGCL 3/0, a resorbable monofilament suture, as both are frequently used in the context of oral surgery. We aimed to investigate the changes in their strength in the presence of tea, coffee, and cola—beverages that such sutures are frequently exposed to in the mouth—and in terms of varying waiting times. Similar studies in the literature have shown that the strength of suture materials decreases over time in a dry environment [11]. To eliminate this situation, many researchers have used saline or artificial saliva; however, saline solution does not have the same chemical properties as saliva. Previous studies have also reported different effects on the strength of suture materials [15]. As natural human saliva is not easy to collect and store, artificial saliva is preferable when attempting to create an environment close to that of the oral environment.

There are many factors affecting suture strength in the oral cavity, one of the most important of which is temperature changes. The oral region is regularly exposed to temperature changes, which can cause the expansion and contraction of suture materials in the mouth and change their physical properties. For this reason, we aimed to prepare the closest model to the oral environment through the application of a thermal aging technique to all test materials. In vitro studies involving the use of thermal aging procedures have achieved similar results to clinical studies [16]. Most researchers report that 20–50 temperature cycles occur per day [17]. Therefore, in our study, we applied thermal aging by applying 40 cycles per day. To the best of our knowledge, our study is the first study in which a thermal aging technique was applied while examining the effects of different beverages on the strength of suture materials.

The pH of the medium was adjusted according to the findings of the study by Chu et al. [18], as the pH of the medium in contact with the suture material plays an important role in its deterioration. The pH of the artificial saliva was adjusted to between 7.4 and 8.1 through continuous monitoring and complete replacement when changes in pH were observed, following studies in the literature [4].

Another important factor in the studies conducted to measure the strength of suture materials is the calibration of the device that applies the tensile force. The universal testing machine used in our study was calibrated according to the guidelines set by Kim et al. [19]. In addition, the type and technique of suture knotting is also important in terms of tensile strength [20,21]. In our study, a simple suturing technique and surgeon’s knots were used as recommended, in order to prevent unraveling [11]. In addition, the test duration in our study was set as 3, 7, and 14 days after evaluating the test durations of similar studies in the literature [10,11]. The duration of contact of the suture materials with beverages during the day was also determined with reference to the study by Abullais et al. [10].

According to the tensile strength results, the strength of the PGCL suture material was significantly higher than that of PGLA at the beginning, as well as at days 3 and 7. At day 14, the strength of both suture materials had decreased significantly, but PGCL exhibited a more dramatic decrease than PGLA and showed similar strength values. These results are in parallel with the study of Alsarhan et al. [11]. This can be explained by the fact that multi-filament sutures have a multi-filament braided structure and are more resistant to hydrolytic degradation compared to monofilament sutures [19]. It is also noteworthy that, although there was a significant loss of resistance in the suture materials at the end of 14 days, no dissolution was observed.

Hans et al. examined the effects of different beverages on salivary pH and reported that cola and coffee significantly lowered the salivary pH and maintained their corrosive properties for a longer period of time [6]. Consequently, their effects on the strength of suture materials are also important. In our study, it is thought that the loss of strength in the suture materials was due to the fact that they remained at a low pH for a longer time depending on the contact time with the liquids in which they are immersed, in addition to the elapsed time. Especially in the case of the PGCL suture material, we believe that cola significantly decreased the resistance at T1 and T2, compared to other beverages.

Ferguson and Khiste have reported that the strength of Vicryl decreased when soaked in saliva, compared to other fluids such as soy, saline, or milk. They stated that saliva increased the degradation of sutures, which resulted in decreased strength. In another in vitro study conducted by Khiste et al. [22], Vicryl sutures showed a decrease in tensile strength under simulated oral conditions. They maintained their resistance until days 7 and 10, and presented minimum strength at day 14. The results obtained in our study paralleled those of Ferguson and Khiste.

In the case of the PGCL suture material, unlike PGLA, the cola beverage significantly decreased its strength, compared to artificial saliva and tea at day 3 and compared to artificial saliva and coffee at day 7.

The current in vitro experimental design has some limitations, and the results obtained may not be fully applicable to the clinical scenario. Clinical studies on the subject will be useful. Substances such as caffeine in tea, coffee, and cola may also cause changes in the structure of sutures through different chemical reactions. The outcome of the study may also be influenced by other factors such as dietary habits, oral hygiene, diseases, and medications, in addition to beverages that can potentially alter the pH level of saliva and cause changes in the strength of suture materials. This article showed that beverages significantly reduced the strength of suture materials for 14 days after surgery. In particular, cola decreased the strength of PGCL suture material more markedly than other beverages in the first week. The exact mechanism by which these beverages cause a decrease in the tensile strength of suture materials remains unclear. Therefore, additional clinical and molecular studies, as well as scanning electron microscopy investigations, are needed. In addition, although the study was carried out with different beverages, the suture materials were always kept in the same pH environment. Therefore, additional studies involving different pH environments should be carried out. Additionally, other variables such as microbiological assessment [23] and anti-infective biocoating [24] should be evaluated in future reports. 

## 6. Conclusions

The study described in this paper demonstrated that beverages significantly decreased the strength of suture materials for 14 days after surgery. In particular, cola decreased the resistance of the PGCL suture material more significantly than other beverages in the first week. Therefore, it is recommended that patients be advised to reduce their consumption of these beverages, especially cola, during the recovery period following surgery. It is also worth noting that the tension loss in the PGCL suture material at the end of the 14-day period was higher than that observed for the PGLA material.

## Figures and Tables

**Table 1:** Tension levels of PGCL and PGLA suture materials according to fluids at each measurement time. Lowercase: The change between times in each fluid for PGCL and PGLA suture materials. Uppercase: The change between fluids for PGCL and PGLA suture materials at each time point.

		Tensile Strength		
	T0 (Day 0)	T1 (Day 3)	T2 (Day 7)	T3 (Day 14)
**PGCL**				
Artificial saliva (*n* = 13)	201.41 ± 10.71 ^a,A^	176.54 ± 17.33 ^b,A^	166.20 ± 19.13 ^b,A^	77.28 ± 14.96 ^c,A^
Tea (*n* = 13)	202.91 ± 11.14 ^a,A^	159.59 ± 20.85 ^b,AB^	149.27 ± 20.10 ^b,AB^	73.13 ± 15.84 ^c,A^
Coffee (*n* = 13)	203.87 ± 11.95 ^a,A^	150.95 ± 20.30 ^b,BC^	158.58 ± 21.69 ^b,A^	69.54 ± 13.18 ^c,A^
Cola (*n* = 13)	204.67 ± 13.31 ^a,A^	133.24 ± 11.74 ^b,C^	130.46 ± 14.77 ^b,B^	74.06 ± 14.69 ^c,A^
**PGLA**				
Artificial saliva (*n* = 13)	121 ± 13.35 ^a,A^	122.16 ± 13.92 ^a,A^	106.53 ± 13.14 ^a,A^	75.72 ± 10.46 ^b,A^
Tea (*n* = 13)	123.61 ± 11.14 ^a,A^	111.24 ± 9.87 ^a,A^	101.27 ± 11.58 ^a,A^	75.88 ± 12.33 ^b,A^
Coffee (*n* = 13)	116.56 ± 13.85 ^a,A^	116.05 ± 9.86 ^a,A^	101.66 ± 7.50 ^a,A^	69.48 ± 7.74 ^b,A^
Cola (*n* = 13)	121.55 ± 12.11 ^a,A^	103.85 ± 5.37 ^ab,A^	97.45 ± 6.83 ^b,A^	58.22 ± 9.60 ^c,A^

## Data Availability

The data set is available upon request from the authors.
